# Primary Breast Mucosa-Associated Lymphoid Tissue (MALT) Lymphoma Transformation to Diffuse Large B-cell Lymphoma: A Case Report

**DOI:** 10.5152/tjh.2011.80

**Published:** 2012-10-05

**Authors:** Şerife Hülya Arslan, Ümmügül Üyetürk, Emre Tekgündüz, Sultan Çiğdem Irkkan, Meltem Yüksel Kurt, Itır Şirinoğlu Demiriz, Fevzi Altuntaş

**Affiliations:** 1 Dr. Abdurrahman Yurtarslan Oncology Education and Research Hospital, Department of Hematology, Ankara, Turkey; 2 Dr. Abdurrahman Yurtarslan Oncology Education and Research Hospital, Department of Medical Oncology, Ankara, Turkey; 3 Dr. Abdurrahman Yurtarslan Oncology Education and Research Hospital, Department of Pathology, Ankara, Turkey

**Keywords:** Primary breast mucosa-associated lymphoid tissue (MALT) lymphoma, Diffuse large B-cell lymphoma

## Abstract

Primary non-Hodgkin’s lymphoma (NHL) of the breast constitutes 0.04%-0.53% of all malignancies and 2.2% of extra nodal lymphomas. In total, 7%-8% of all B-cell lymphomas are the mucosa-associated lymphoid tissue (MALT) type, of which up to 50% of primary gastric MALT lymphoma. Herein we present a patient with breast MALT lymphoma that transformed to diffuse large B-cell lymphoma (DLBCL). A 69-year-old female presented with a mass on her left breast. Physical examination showed a 3×3-cm mass located 1 cm from the areola on the upper lateral quadrant of the breast at the 1 o’clock position, which was fixed and firm. Excisional biopsy was performed and pathologic examination of the specimen showed MALT lymphoma transformation to DLBCL. The patient was staged as II-EA. The rituximab, cyclophosphamide, doxorubicin, vincristine, and prednisolone (R-CHOP) protocol was scheduled as treatment. Following 6 courses of R-CHOP, 2 additional courses of rituximab were administered. Positron emission tomography (PET)-CT was done at the end of the treatment. PET showed that the patient was in complete remission. At the time this report was written, the patient was being followed-up at the outpatient clinic on a regular basis. Lymphoma of the breast is a rarity among malignant tumors of the breast. The most common type of lymphoma is DLBCL. Breast MALT lymphoma is extremely rare. Primary MALT lymphoma of the breast can transform from low grade to high grade and recurrence is possible; therefore, such patients should be monitored carefully for transformation.

## Abstract (tr)

Memenin primer non-Hodgkin lenfomaları (NHL), meme dokusu malignitelerinin %0.04-0.53’ünü; ekstranodal lenfomaların ise %2.2’sini oluşturmaktadır. B hücreli lenfomaların %7-8’ini mukoza ilişkili lenfoid doku (mucosaassociated lymphoid tissue-MALT) tipi lenfomalar, bunların da %50’den fazlasını primer gastrik MALT lenfomalar oluşturmaktadır. Burada memede nadir görülen MALT lenfoma zemininde diffüz büyük B hücreli lenfomaya (DBBHL) dönüşen bir olgunun sunulması amaçlanmıştır. Sol memede şişlik şikayeti ile başvuran 69 yaşındaki kadın hastanın yapılan muayenesinde, sol meme üst dış kadranda saat 1 hizasında, areoladan 1 cm uzaklıkta 3x3 cm boyutlarında sert kitle tespit edildi. Yapılan eksizyonel biyopsisinin patolojik incelemesinde DBBHL’ya progresyon gösteren MALT lenfoma tanısı konuldu. Hasta evre II EA olarak evrelendi. Hastaya rituksimab, siklofosfamid, doksorubisin, vinkristin, prednizolon tedavisi planlandı (R-CHOP). Hastaya 6 kür R-CHOP ve sonrasında 2 kür R verildi. Yanıt değerlendirmesi için çekilen pozitron emisyon tomografisi (PET)-BT ile tam remisyon sağlandığı görüldü. Halen hastanın takibine devam edilmektedir. Memenin malign tümörleri içerisinde meme lenfomaları nadir görülmektedir. Meme lenfoma olgularının en sık görülen alt tipi DBBHL’dır. Memede MALT lenfoma ise çok daha nadir görülmektedir. Memenin primer MALT lenfomasının düşük dereceli lenfomadan, yüksek dereceliye dönüşüm ve nüks olasılığı nedeniyle dikkatli takibi gerekmektedir.

## INTRODUCTION

Primary, non-epithelial breast tumors constitute 5% of all malignancies originating from breast, and must be included in the differential diagnosis of breast masses. In patients with lymphoma of the breast secondary breast involvement occurs often, although primary breast lymphoma is extremely rare [1]. Primary non-Hodgkin’s lymphoma (NHL) of the breast constitutes 0.04%-0.53% of all malignancies and 2.2% of extranodal lymphomas [2]. Lymphocytes in breast tissue are located close to the axillary region, upper lateral quadrant, in lymph nodes, and in the lymphatic ductus. These lymphoid aggregations are the major cause of lymphoid neoplasia [3]. Informed consent was obtained. 

In all, 7%-8% of B-cell lymphomas are the mucosaassociated lymphoid tissue (MALT) type, of which up to 50% of primary gastric MALT lymphoma. Furthermore, during the course of autoimmune diseases MALT lymphomas often involve such tissues as skin, ocular adnexal, lung, salivary gland, thyroid, and breast [4]. Herein we present a case with primary breast MALT lymphoma that transformed to diffuse large B-cell lymphoma (DLBCL).

## CASE

A 69-year-old female presented with a mass on her left breast, which was first noticed approximately 3 months earlier. Physical examination showed a 3 x 3-cm mass located 1 cm from the areola on the upper lateral quadrant of the breast at the 1 o’clock position, which was fixed and firm. The patient did not have sweats, weight loss, or fever. Her medical history was unremarkable. Complete blood count was normal, biochemistry was normal, except for LDH of 274 U L^-1^ (normal range: 0-200 U L^-1^), and the erythrocyte sedimentation rate (ESR) was 56 mm h^-1^.

**Figure 1:**
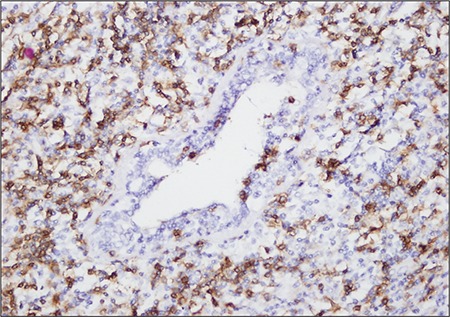
Small lymphoid cells infiltrating along the LCA andbreast ducts, forming aggregates around the ducts (400x).

**Figure 2:**
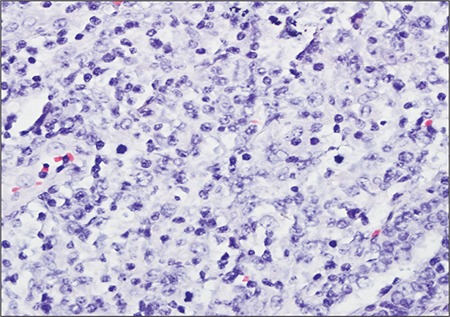
Diffuse infiltration pattern of the tumor removed fromthe breast tissue. Frequent mitosis and apoptosis can be seeninside the diffuse large B-cell lymphoma region, in which largecentroblastic lymphoid cells are observed (HE, 400x)

Excisional biopsy was performed and pathologic examination of the specimen showed a lymphoepithelial lesion rich in plasma cells, centroblasts, and immunoblast-like cells. These regions stained diffusely with LCA (Figure 1), CD20, and CD79a, and focally with bcl2. An extensive invasion pattern had destroyed the breast tissue; frequent mitosis and apoptosis were observed. Large centroblastictype and polymorphic lymphoid cells were interpreted as MALT lymphoma transforming to DLBCL (Figure 2). 

Analysis of a bone marrow biopsy specimen was negative for lymphoma infiltration. Cervical and abdominal computed tomography (CT) showed no involvement. Thoracic CT showed a 15-mm lymphadenopathy in the left axillary zone. The patient was staged as II-EA. The rituximab (375 mg m-²) cyclophosphamide (750 mg m-²), doxorubicin (50 mg m-²), vincristine (1.4 mg m-² [maximum: 2 mg]), methylprednisolone (80 mg d^-1^) (R-CHOP) protocol was scheduled as the treatment. After 6 courses of R-CHOP, 2 additional courses of rituximab were administered. Positron emission tomography (PET-CT) was done at the end of the treatment. PET showed that the patient was in complete remission.

## DISCUSSION

Primary breast lymphomas present in elderly woman as painless, unilateral masses. Most cases are consistent with B-cell NHL. The most common subtypes are DLBCL and marginal zone lymphoma [5]. Prognosis depends on histological subtype and stage. Stage I disease is treated with involved-field radiation only. Marginal zone lymphomas rarely transform to the aggressive DLBCL [6,7]. 

Median age at the time primary breast lymphoma is diagnosed is 60 years, although high-grade lymphomas are seen in patients younger than 60 years [8]. Diagnostic criteria include involvement of breast and lymphoid tissue in the tumor, negative history of extra-mammary lymphoma, absence of extensive lymphoma involvement, and the breast as the primary region of involvement [9]. The presented case had simultaneous pathological breast and lymphoid tissue involvement. Therefore the primary focus of lymphoma is a matter of speculation. Left axillary lymph node involvement was interpreted as adjacent lymph node extension, as the mass was located within the left breast. 

Fine needle aspiration is often unsuccessful in diagnosing such cases; therefore, as in the presented case, excisional biopsy should be performed [10]. Histologically, MALT lymphomas invade epithelial tissues and exhibit proliferation of neoplastic marginal zone cells that form the characteristic lymphoepithelial lesions [11]. MALT lymphomas are low-grade lymphomas and rarely progress to high-grade, which occurs in only 10% of cases and is related to such genetic abnormalities as p16INK and p53 activation [11]. 

Primary breast MALT lymphomas generally tend to be limited; therefore, mastectomy is not recommended, except in cases with bulky disease and aggressive histopathology; breast-conserving surgery is the preferred treatment method. Although the present case had stage II disease, transformation to aggressive lymphoma indicated that systemic treatment was the best option, which resulted in complete remission and complication-free follow-up. 

Differential diagnosis of breast masses includes lymphomas, although they are extremely rare. Primary MALT lymphoma of the breast can transform from low grade to high grade, and careful patient monitoring is necessary because of the risk of recurrence. 

## CONFLICT OF INTEREST STATEMENT

The authors of this paper have no conflicts of interest, including specific financial interests, relationships, and/ or affiliations relevant to the subject matter or materials included.
